# Influence of Soft Stabilization Splint on Electromyographic Patterns in Masticatory and Neck Muscles in Healthy Women

**DOI:** 10.3390/jcm12062318

**Published:** 2023-03-16

**Authors:** Grzegorz Zieliński, Marcin Wójcicki, Michał Baszczowski, Agata Żyśko, Monika Litko-Rola, Jacek Szkutnik, Ingrid Różyło-Kalinowska, Michał Ginszt

**Affiliations:** 1 Department of Sports Medicine, Medical University of Lublin, 20-093 Lublin, Poland; 2 Independent Unit of Functional Masticatory Disorders, Medical University of Lublin, 20-093 Lublin, Poland; 3 Interdisciplinary Scientific Group of Sports Medicine, Department of Sports Medicine, Medical University of Lublin, 20-093 Lublin, Poland; 4 Department of Dental and Maxillofacial Radiodiagnostics, Medical University of Lublin, 20-093 Lublin, Poland; 5 Department of Rehabilitation and Physiotherapy, Medical University of Lublin, 20-093 Lublin, Poland

**Keywords:** stabilization splint, surface electromyography, temporalis, masseter, digastric, sternocleidomastoid, functional indices

## Abstract

This study investigates the influence of soft stabilization splints on electromyographic patterns in masticatory and neck muscles in healthy women. A total of 70 healthy women were qualified for the research. The resting and clenching electromyographic patterns of the temporalis (TA), masseter (MM), digastric (DA), and sternocleidomastoid (SCM) muscles were measured using the BioEMG III™ apparatus. The interaction between splint application and resting muscle activity affected the results in all examined muscles except the temporalis muscle. A large effect size was observed in masseter (2.19 µV vs. 5.18 µV; *p* = 0.00; ES = 1.00) and digastric (1.89 µV vs. 3.17 µV; *p* = 0.00; ES = 1.00) both-sided RMS activity. Significant differences between the two conditions were observed in all Functional Clenching Indices (FCI) for MM, SDM, and DA muscles. All FCI values for the MM and DA muscles were significantly lower with than without the splint. We observed an increase in all activity indices due to splint application, which suggests a masseter muscle advantage during measurement. The soft stabilization splint influenced resting and functional activity in the MM, SDM, and DA muscles. During tooth clenching, a soft stabilization splint changed the involvement proportions of the temporalis and masseter muscles, transferring the main activity to the masseter muscles. Using a soft stabilization splint did not affect the symmetry of the electromyographic activity of the masticatory and neck muscles.

## 1. Introduction

Managing dysfunctions of the stomatognathic system requires a comprehensive multidirectional approach, and is still challenging for clinicians worldwide. Temporomandibular disorders (TMDs) are the most common form of non-odontogenic orofacial pain, and drastically reduce life quality [1]. It has been estimated that 4% of adults develop clinically confirmed and painful TMDs each year. Moreover, the occurrence of TMDs increases with age, with peak incidence being reported as 4.5% in the 35–44 age group [2]. Treatment options for patients with TMDs, bruxism, and frequent headaches associated with stomatognathic system disorders include pharmacotherapy [3], physiotherapy [4], patient education [5], behavior therapy [6], and removable appliances called occlusal or stabilization splints [7]. There are many types of splints varying in design, e.g., the material from which they are made (hard and soft splints), the position of the splint (maxillar and mandibular), and the extent of coverage (full-arch-covering type and partial type covering only the central incisors) [8,9]. The effectiveness of the use of stabilization splints, both soft and hard, has been demonstrated in various scientific reports. Stabilization splint therapy has been described as a well-established treatment for TMDs [10,11], bruxism [12,13], and headache [14]. Soft stabilization splints are effective in the symptomatic management of TMDs, especially for symptoms such as temporomandibular joint (TMJ) clicking, TMJ pain, and masticatory muscle pain [15]. Moreover, stabilization splint therapy may reduce pain severity at rest and on palpation in patients with temporomandibular myofascial pain [16]. Therefore, occlusal splint therapy seems to be an efficient treatment for TMD patients, as proved by several studies with a success rate of 70–90% [17]. In addition, stabilization splints are used to control bruxism, prevent tooth abrasion, and stimulate muscular relaxation [12].

On the other hand, the effectiveness of stabilization splints is controversial. A systematic review found that a hard stabilization splint does not appear more effective than a soft splint, a non-occluding palatal splint, or physical therapy for managing masticatory muscle pain [18]. Based on a randomized controlled trial, stabilization splint treatment in combination with counseling and masticatory muscle exercises does not offer any additional benefit in relieving masticatory muscle pain and increasing mandible mobility compared to counseling and masticatory muscle exercises alone over a short time interval [19]. In addition, the positive effect of a stabilization splint on signs and symptoms of TMDs could not be confirmed or refuted based on a systematic review [20]. Moreover, soft splints show some disadvantages. A soft occlusal splint can encourage muscle hyperactivity, and deteriorates more quickly than a hard splint [21,22]. It has been reported that occlusal stabilization splints increased surface electromyographic (sEMG) values during clenching activity [23,24]. Despite many studies in this area, the clinical effectiveness of soft stabilization splints remains uncertain [7,11,20]. Moreover, the way a splint affects the proportions of the activity of the temporalis muscle and the masseter muscle, and whether it affects the activity of the cervical spine muscles, have yet to be clarified. The way a splint affects the activity of antagonist muscles, both at rest and during tooth clenching, also requires explanation. Despite many questions and a lack of clear scientific evidence for the healing effect of soft stabilization splints in bruxism and TMDs management, they are widely used in clinical practice.

Therefore, this study investigates the influence of soft stabilization splints on electromyographic patterns in the masticatory and cervical spine muscles of healthy women. We decided to apply sEMG measurement because it is commonly used in dentistry to analyze the myoelectric signals of masticatory muscles [25]. Physiological variations in the state of muscle fiber membranes form myoelectric signals. Surface electrodes permit noninvasive measurement of bioelectrical phenomena of muscular activity [26]. The interpretation of sEMG records involves using electromyographic indices to increase the validity of electromyographic examination [27]. Using standardized and novel functional indices for masticatory and neck muscle activity, and the assessment of four muscle groups, this study aimed at a complete analysis of bioelectrical activity when a soft stabilization splint is applied. We assumed that the splint would affect the resting and functional activity of the masticatory muscles in functional, activity, and asymmetry indices. In addition, we assumed that applying the soft stabilization splint would affect the activity of the masticatory antagonistic muscles and the cervical spine muscles.

## 2. Materials and Methods

### 2.1. Study Population

Ethical approval was obtained from the Bioethical Committee of the Medical University of Lublin (KE-0254/81/2021). The objectives and methods of the study were fully explained to the participants, who provided written informed consent. The Strengthening the Reporting of Observational Studies in Epidemiology (STROBE) inventory was used to evaluate research quality [28]. The participants were 70 healthy young women (mean age 23.4 ± 2.2 years). Recruitment and measurements for the presented research were conducted at the Medical University of Lublin (Independent Unit of Functional Masticatory Disorders) between November 2021 and July 2022. The inclusion criteria were the following: (a) female gender; (b) age between 18 and 35 years; and (c) absence of temporomandibular disorders (TMDs), which was assessed using the Research Diagnostic Criteria for Temporomandibular Disorders (RDC/TMD) protocol. Women with any of the following were excluded from the project: any temporomandibular disorders (e.g., temporomandibular joint pain, masticatory muscle pain, disc displacement, temporomandibular joint diseases), any pain condition within the stomatognathic system, fibromyalgia, regular headaches, Angle’s Class II or III malocclusion, open bite, lack of at least four support zones in dental arches, lack of more than four teeth within both dental arches, periodontal diseases, orthodontic treatment, possession of dental prostheses, neurological disorders, history of Botulinum toxin therapy, or current pregnancy. Moreover, an ultrasound examination was conducted using an M-Turbo ultrasound device (SonoSite, Inc., Bothell, WA, USA) to assess the condition of subjects’ temporomandibular joint and masticatory muscles.

### 2.2. Oral Appliance Design

**Figure 1:**
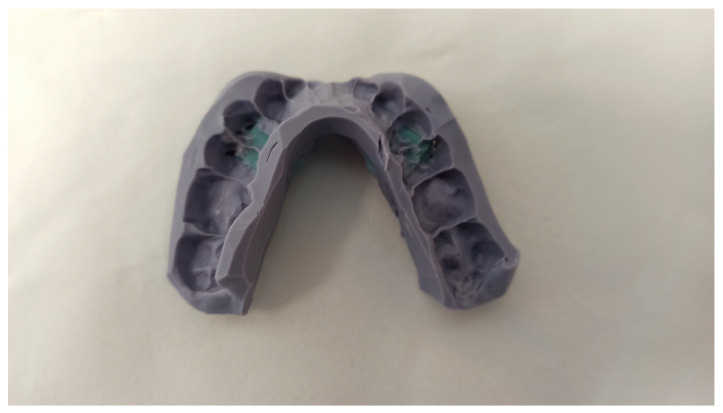
Soft stabilization splint example.

The study protocol consisted of two phases: (1) non-splint measurement; (2) splint measurement. A random choice was performed for the initial measurement. The women completed two masticatory tasks (resting activity and tooth clenching) with and without soft stabilization splints between the teeth. Soft stabilization splints were fabricated directly in the women’s mouths using Variotime^®^ Easy Putty material [23,29]. Each splint was 4 mm thick, measuring between the upper and lower premolars. The base and catalyst of the silicone material were mixed by hand according to the manufacturer’s directions and formed into a cylinder. The material was then placed onto the patient’s lower arch covering every tooth. To ensure proper thickness, two 2-mm thick Fleximeter^®^ Strips were placed on both sides of the cylinder in the premolar region. Then the patient was asked to close her mouth until the teeth touched the Fleximeter^®^ Strips [10,23]. The excess of each splint was cut off using a scalpel to provide maximum comfort to the patient while maintaining a stable position for the mandibular and maxillary arches (Figure 1).

### 2.3. Electromyographic Examination

Resting (10 s) and maximum-clenching (3 times for 3 s, with 2 s break) bioelectric activity of the temporalis anterior (TA), superficial masseter (MM), anterior belly of the digastric muscle (DA), and middle part of the sternocleidomastoid muscle (SCM) was recorded using an 8-channel sEMG device (BioEMG III™, BioResearch Associates, Inc., Milwaukee, WI, USA). During electromyographic measurement, the participant sat in a dental chair with her head on the headrest and her torso perpendicular to the ground. Surface electrodes (Ag/AgCl, 30 mm diameter, 16 mm conductive surface, SORIMEX, Toruń, Poland) were placed bilaterally following the course of the fibers of the muscles, as previously described [30]. 

Microvolt values were amplified with minimal noise to 5000 times their original levels. Moreover, the sEMG values were reduced by 40 dB with a Noise Buster filter, eliminating 99% of 50/60 Hz sEMG noise. The electromyographic potentials based on root mean square (RMS) calculations were used to obtain the mean sEMG outcomes.

Mean RMS TA (TA tot) = (TA-R + TA-L)/2(1)

Mean RMS MM (MM tot) = (MM-R + MM-L)/2(2)

Mean RMS SCM (SCM tot) = (SCM-R + SCM-L)/2(3)

Mean RMS DA (DA tot) = (DA-R + DA-L)/2(4)

Functional Clenching Indices (FCI), Functional Clenching Activity Indices (FCAI), and Functional Clenching Symmetry Indices (FCSI) were used to normalize the mean bioelectric potentials. Indices were calculated based on mean RMS clenching (CL) and resting (REST) activity, according to our previous sEMG protocol [27]:

FCI for TA right-sided (FCI TA-R) = CL TA-R/REST TA-R(5)

FCI for TA left-sided (FCI TA-L) = CL TA-L/REST TA-L(6)

FCI for TA both-sided (FCI TA tot) = (CL TA-R + CL TA-L)/(REST TA-R + REST TA-L)(7)

FCI for MM right-sided (FCI MM-R) = CL MM-R/REST MM-R(8)

FCI for MM left-sided (FCI MM-L) = CL MM-L/REST MM-L(9)

FCI for MM both-sided (FCI MM tot) = (CL MM-R + CL MM-L)/(REST MM-R + REST MM-L)(10)

FCI for SCM right-sided (FCI SCM-R) = CL SCM-R/REST SCM-R(11)

FCI for SCM left-sided (FCI SCM-L) = CL SCM-L/REST SCM-L(12)

FCI for SCM both-sided (FCI SCM tot) = (CL SCM-R + CL SCM-L)/(REST SCM-R + REST SCM-L)(13)

FCI for DA right-sided (FCI DA-R) = CL DA-R/REST DA-R(14)

FCI for DA left-sided (FCI DA-L) = CL DA-L/REST DA-L(15)

FCI for DA both-sided (FCI DA tot) = (CL DA-R + CL DA-L)/(REST DA-R + REST DA-L)(16)

FCAI right-sided (FCAI-R) = (FCI MM-R − FCI TA-R)/(FCI MM-R + FCI TA-R) × 100(17)

FCAI left-sided (FCAI-L) = (FCI MM-L − FCI TA-L)/(FCI MM-L + FCI TA-L) × 100(18)

FCAI both-sided (FCAI tot) = (FCI MM tot − FCI TA tot)/(FCI MM tot + FCI TA tot) × 100(19)

FCSI TA = (FCI TA-R − FCI TA-L)/(FCI TA-R + FCI TA-L) × 100(20)

FCSI MM = (FCI MM-R − FCI MM-L)/(FCI MM-R + FCI MM-L) × 100(21)

FCSI SCM = (FCI SCM-R − FCI SCM-L)/(FCI SCM-R + FCI SCM-L) × 100(22)

FCSI DA = (FCI DA-R − FCI DA-L)/(FCI DA-R + FCI DA-L) × 100(23)

The following formulas were used to calculate activity (ACI) and asymmetry (ASI) indices based on the mean RMS potentials recorded during resting and functional activity, as specified by Naeije et al. and Ferrairo et al. [31,32]:

ACI right-sided (ACI-R) = (MM-R − TA-R)/(MM-R + TA-R) × 100(24)

ACI left-sided (ACI-L) = (MM-L − TA-L)/(MM-L + TA-L) × 100(25)

ACI both-sided (ACI tot) = (MM-R + MM-L − TA-R − TA-L)/(MM-R + MM-L + TA-R + TA-L) × 100(26)

ASI TA = (TA-R − TA-L)/(TA-R + TA-L) × 100(27)

ASI MM = (MM-R − MM-L)/(MM-R + MM-L) × 100(28)

ASI SCM = (SCM-R − SCM-L)/(SCM-R + SCM-L) × 100(29)

ASI DA = (DA-R − DA-L)/(DA-R + DA-L) × 100(30)

### 2.4. Statistical Calculations

The repeatability of the sEMG procedure was verified with duplicate sEMG examinations on 10 participants, as previously reported [33]. An analysis of power was conducted using the G*Power 3.1.9.7 program (Heinrich Heine University Düsseldorf, Germany) [34]. The sample size was calculated based on previous studies [27]. The calculations indicated that a sample size of 68 participants would be sufficient to notice a significant difference between two independent means (*t*-test) with an *α* value of 0.05, a power value of 0.90, and an estimated medium effect size of 0.56.

The data comparison was performed using the GraphPad Prism 9.4.1 program (GraphPad Software, Inc., San Diego, CA, USA). The normality of the distribution of variables was verified using the Shapiro–Wilk test and the Kolmogorov–Smirnov test (with the Lilliefors correction). The Student’s *t*-test (T) or Mann–Whitney U test (Z) was used to compare the differences between groups. The results were presented in the form of minimum (Min), maximum (Max), mean, and standard deviation values (SD). Effect sizes were determined for the *t*-test using the Cohen d method and interpreted as small (0.2), medium (0.5), or large (0.8) effect sizes [35,36]. A confidence interval (CI 95%) was calculated for results at a level of 95% [37]. Statistical significance was set at *p* ≤ 0.05.

## 3. Results

Based on the study criteria, 70 women were qualified for the research. The participants’ general characteristics, including age, height, weight, and mandibular range of motion, are presented in Table 1. 

The interaction between splint application and resting muscle activity affected the results within all examined muscles except the temporalis muscle (Table 2). A large effect size was observed in masseter (2.19 µV vs. 5.18 µV; *p* = 0.00; ES = 1.00) and digastric (1.89 µV vs. 3.17 µV; *p* = 0.00; ES = 1.00) both-sided RMS activity. In all cases, the splint application caused a significant increase in the resting RMS activity of the examined muscle groups. However, significant differences were observed only for the masseter and sternocleidomastoid muscle during clenching activity, with a small effect size (Table 3). In these measurements, an increase in the functional activity of the examined muscles was also observed after placing the stabilization splint.

Table 4 shows significant differences between the two conditions in all functional clenching indices for masseter, sternocleidomastoid, and digastric muscles. All FCI values for the masseter (small effect size) and digastric muscles (large effect size) were significantly lower with than without the splint. The opposite tendency was determined in the sternocleidomastoid muscle, where splint application caused an increase in functional clenching indices with large effect sizes in the right and left muscle groups. We also observed a significant decrease in functional clenching activity indices for the right and both-sided values with small effect sizes. Significant differences between the two measurements were observed in all activity indices in resting and clenching conditions with small to medium effect sizes (Table 5). In all cases we observed increased activity indices due to splint application, which suggests a masseter muscle advantage during measurement. 

## 4. Discussion

This investigation aimed to examine the influence of soft stabilization splints on electromyographic patterns in masticatory and neck muscles in healthy women. We assumed that the splint would affect resting and functional activity in the masticatory muscles. In addition, we thought that applying the soft stabilization splint would affect the activity of the masticatory antagonistic muscles and the cervical spine muscles. Using hard stabilization splints seems more reasonable than soft splints for TMDs and bruxism. In several reports, hard and soft stabilization splints effectively treat TMDs. However, hard stabilization splints provide a quicker decrease in TMD symptoms [15]. Moreover, compared to the absence of a splint, hard splints produced less strain on molar teeth but more strain on premolar teeth during maximum voluntary tooth clenching. In contrast, soft occlusal splints did not reduce the strain on all target teeth significantly during clenching tasks [38]. Hard stabilization splints significantly reduced the number of sEMG high-activity events per hour of sleep. In contrast, soft occlusal splints do not inhibit jaw muscle activity compared to baseline values [39]. Despite the advantage of a hard splint, soft splints are used to ease implementation and reduce financial costs to the patient [7]. However, the effect of using soft splints on masticatory muscle activity has yet to be unequivocally evaluated in current studies.

This study showed increased functional activity during tooth clenching in splint condition and increased resting activity of masticatory muscles during splint application within RMS values. The increased resting electromyographic RMS activity was significant in the masseter, digastric, and sternocleidomastoid muscle groups. The increase in the RMS values was also detectable in the temporalis muscle at rest, but the differences were not statistically significant. This may be related to lower reaction of the temporalis muscle to the mandibular position change compared to the other muscle groups. Applying the splint did not change the asymmetry of the masticatory muscles at rest in ASI indices. The Gholampour et al. study showed a considerable symmetry effect in splint condition [13]. In the abovementioned investigation, the splint allowed for asymmetric and non-uniform loading. However, the splints were produced using a hard polymerized colorless acrylic resin, compared to the silicone material used in our investigation. Splint application significantly affected the ratio of the temporalis muscle’s involvement with the masseter muscle in ACI indices. In resting activity and during tooth clenching, a soft stabilization splint changed the involvement proportions of the temporalis and masseter muscles, transferring the main activity to the masseter muscles. The functional RMS activity during tooth clenching with the soft splint was significantly higher in the masseter and the sternocleidomastoid muscle than without the splint. This may indicate the need for greater stabilization of the cervical spine during clenching due to the phenomenon of co-contraction [40]. The results of the Akat et al. study identified significant differences in sEMG parameters with hard and soft occlusal splints after three months of treatment [12]. In the study, sEMG activity decreased with all splint types, most prominently in the hard occlusal splint group. The differences in our observations may result from a different population (subjects with diagnosed bruxism, mixed population vs. healthy young women), a more extended observation period (3 months vs. immediate effect), and methodological assumptions of the sEMG signal analysis (raw sEMG signal vs. sEMG normalization). Our experiment used RMS data, validated ASI and ACI indices, and Functional Indices for the electromyographic assessment. We observed a significant decrease for all Functional Clenching Indices and Functional Clenching Activity Indices in the masseter, sternocleidomastoid, and digastric muscle groups. This decrease may indicate a disturbed proportion between the resting and functional activity of the examined muscles [27]. However, the soft stabilization splint did not affect the results in terms of asymmetry indices in Functional Clenching Symmetry Indices. Therefore, the proportions of masticatory muscle involvement at rest and during activity on the left and right sides do not seem to change under stabilization splint application.

The presented research has several limitations that should be addressed in future investigations. Firstly, our results are limited by the immediate follow-up. Secondly, in our study, each splint was 4 mm thick, measuring between the upper and lower premolars. According to the current report, 2 mm and 4 mm splints effectively treat muscle disorders and disc displacements, especially for masticatory muscle pain and TMJ acoustic symptoms [10]. Therefore, we decided on a standard size for all patients to standardize the measurement results. In temporomandibular disorder therapy, a splint that is individually adjusted to the current resting position of the mandible and minimal resting activity of the masticatory muscles should be used. Thirdly, we tested our splint on healthy adult women. It was estimated that TMDs affect women more often than men [41,42]. Moreover, we standardized our study group to eliminate the influence on the results of the experiment of gender, age, and changes in muscle activity patterns in response to pain. Therefore, future research should be performed in patients with stomatognathic system disorders, e.g., TMDs, or bruxism populations. 

Finally, we should stress that we are not claiming that a soft stabilization splint is ineffective for treating TMDs and bruxism. In this paper, we have reported the results of the immediate effect of soft splint use on sEMG muscle activity. However, our study indicates the consideration of the adverse effects of long-term use of soft stabilization splints in the treatment of masticatory system disorders, as muscle hyperactivity and changes in electromyographic patterns over a more extended period may cause adverse effects within the stomatognathic system. Therefore, the long-term impact of soft stabilization splint use on masticatory and neck muscle activity requires further research. Moreover, additional objective measurement methods, e.g., computer simulations, can be used to evaluate splint efficiency in further studies [43].

## 5. Conclusions

Soft stabilization splint use influences resting and functional activity within the MM, SCM, and DA muscles. During tooth clenching activity, a soft stabilization splint changes the involvement proportions of the temporalis and masseter muscles, transferring the main activity to the masseter muscles. Using a soft stabilization splint does not affect the symmetry of the electromyographic activity of the masticatory and neck muscles.

## Figures and Tables

**Table 1:** Participants’ general characteristics, including age, height, weight, and mandibular range of motion.

Variable	Min.	Max.	Mean	SD
Age (years)	19.00	34.00	23.43	2.27
Height (cm)	155.00	183.00	167.93	6.90
Weight (kg)	44.00	84.50	59.94	8.32
Active maximum mouth opening (mm)	32.00	62.00	49.77	5.90
Passive maximum mouth opening (mm)	35.00	65.00	52.49	5.83
Active laterotrusion right (mm)	0.00	13.00	9.51	2.17
Active laterotrusion left (mm)	0.00	15.00	9.99	2.35
Active protrusion (mm)	3.00	14.00	9.11	2.38

Min.—minimum; Max.—maximum; SD—standard deviation.

**Table 2:** Comparison of root mean square (RMS) bioelectric resting potentials with and without stabilization splint.

Muscle	Resting RMS Values without Stabilization Splint (µV)				Resting RMS Values with Stabilization Splint (µV)				Test		*p*	ES	CI 95%	
	Min.	Max.	Mean	SD	Min.	Max.	Mean	SD						
TA-R	0.85	10.54	2.46	1.59	0.59	19.42	3.26	2.97	Z	−1.63	0.10	0.16	−0.08	0.75
TA-L	0.80	6.82	2.56	1.46	0.64	13.54	3.12	2.44	Z	−1.02	0.31	0.10	−0.20	0.71
TA tot	0.83	7.39	2.51	1.26	0.66	15.34	3.19	2.58	Z	−1.27	0.20	0.12	−0.14	0.71
MM-R	0.71	5.73	2.11	1.09	0.77	30.30	5.41	5.65	Z	−5.38	0.00 *	0.53	0.90	2.14
MM-L	0.71	7.72	2.27	1.25	0.83	22.28	4.95	4.45	Z	−4.24	0.00 *	0.42	0.61	1.98
MM tot	0.78	5.19	2.19	1.05	0.80	26.25	5.18	4.83	Z	−5.06	0.00 *	1.00	0.83	2.13
SCM-R	0.68	3.34	1.20	0.41	0.90	6.89	1.70	1.00	Z	−4.34	0.00 *	0.43	0.16	0.43
SCM-L	0.59	2.65	1.34	0.42	0.78	7.42	1.75	0.92	Z	−3.64	0.00 *	0.36	0.13	0.44
SCM tot	0.76	2.71	1.27	0.36	0.91	5.48	1.73	0.84	Z	−4.17	0.00 *	0.76	0.18	0.44
DA-R	0.86	6.46	1.92	0.99	1.14	9.08	3.12	1.56	Z	−5.83	0.00 *	0.57	0.67	1.32
DA-L	0.92	7.12	1.86	1.00	1.16	10.80	3.22	1.73	Z	−6.24	0.00 *	0.61	0.74	1.46
DA tot	0.90	6.79	1.89	0.98	1.23	9.94	3.17	1.57	Z	−5.79	0.00 *	1.00	0.76	1.44

RMS—root mean square; Z—Mann–Whitney U test; ES—effect size; CI—confidence interval; Min.—minimum; Max.—maximum; SD—standard deviation; TA—temporalis muscle; MM—masseter muscle; SCM—sternocleidomastoid muscle; DA—digastric muscle; R—right side; L—left side; tot—both-sided; μV—microvolt; *—significant difference.

**Table 3:** Comparison of root mean square (RMS) functional bioelectric potentials during maximal tooth clenching with and without stabilization splint.

Muscle	Clenching RMS Values without Stabilization Splint (µV)				Clenching RMS Values with Stabilization Splint (µV)				Test		*p*	ES	CI 95%	
	Min.	Max.	Mean	SD	Min.	Max.	Mean	SD						
TA-R	14.70	403.80	145.32	84.67	12.20	745.70	149.41	99.99	Z	−0.28	0.78	0.03	−21.40	25.70
TA-L	12.40	319.00	138.70	68.30	5.90	287.50	137.14	59.82	Z	0.14	0.89	0.02	−20.30	23.20
TA tot	22.60	361.40	142.01	73.42	9.05	411.85	143.27	69.99	T	−0.33	0.74	0.03	−18.85	25.50
MM-R	13.70	419.00	149.56	90.64	29.60	387.90	182.56	86.87	Z	−2.58	0.01 *	0.25	9.30	64.20
MM-L	5.10	526.10	147.99	99.49	26.30	463.20	178.23	94.73	Z	−2.15	0.03 *	0.21	3.70	63.10
MM tot	9.40	441.25	148.78	91.75	27.95	391.85	180.40	86.95	Z	−2.09	0.04 *	0.35	7.70	63.95
SCM-R	1.60	42.50	10.43	7.78	2.30	76.40	12.81	9.39	Z	−2.53	0.01 *	0.24	0.60	4.20
SCM-L	1.40	40.70	10.15	7.91	2.50	193.00	15.08	23.97	Z	−2.47	0.01 *	0.27	0.50	4.20
SCM tot	1.50	41.60	10.29	7.49	2.40	106.55	13.94	14.78	Z	−2.72	0.01 *	0.03	0.70	4.20
DA-R	4.10	66.50	21.74	14.25	4.20	58.00	22.52	11.43	Z	−1.12	0.26	0.09	−1.50	5.50
DA-L	4.60	106.00	22.88	18.63	5.80	91.80	23.59	15.97	Z	−0.90	0.37	0.10	−1.80	5.00
DA tot	4.70	77.05	22.31	14.64	5.00	74.90	23.05	12.76	Z	−1.01	0.31	0.03	−1.65	5.00

RMS—root mean square; Z—Mann–Whitney U test; ES—effect size; CI—confidence interval; Min.—minimum; Max.—maximum; SD—standard deviation; TA—temporalis muscle; MM—masseter muscle; SCM—sternocleidomastoid muscle; DA—digastric muscle; R—right side; L—left side; tot—both-sided; μV—microvolt; *—significant difference.

**Table 4:** Comparison of functional indices with and without stabilization splint.

Indices	Without Stabilization Splint				With Stabilization Splint				Test		*p*	ES	CI 95%	
	Min.	Max.	Mean	SD	Min.	Max.	Mean	SD						
FCI TA-R	4.44	281.80	73.00	51.78	6.74	388.47	67.03	57.52	Z	0.99	0.32	0.10	−19.37	6.29
FCI TA-L	7.87	230.45	70.30	47.74	3.92	280.00	70.41	58.15	Z	0.62	0.53	0.06	−18.68	10.77
FCI tot	8.68	204.60	66.97	42.44	5.41	294.00	65.59	51.18	Z	0.17	0.86	0.03	−16.64	7.89
FCI MM-R	8.09	524.46	90.97	84.91	3.39	239.20	63.92	58.41	Z	2.59	0.01 *	0.25	−33.11	−4.48
FCI MM-L	3.03	395.56	84.64	75.72	3.66	449.71	67.47	73.46	Z	2.08	0.04 *	0.20	−31.31	−1.00
FCI MM tot	5.01	407.87	85.47	75.38	3.77	303.76	61.94	57.68	Z	2.44	0.01 *	0.24	−31.23	−3.05
FCI SCM-R	−24.72	74.01	0.54	10.86	1.85	41.98	8.70	6.35	Z	−9.15	0.00 *	0.94	6.83	8.91
FCI SCM-L	−80.91	24.83	−1.10	10.65	1.45	94.61	8.98	11.54	Z	−9.58	0.00 *	0.96	5.74	8.33
FCI SCM tot	−54.62	10.83	−0.96	7.60	1.62	57.13	8.77	7.89	Z	−9.76	0.00 *	0.25	6.52	8.26
FCI DA-R	1.83	45.50	13.09	9.77	0.01	0.56	0.18	0.10	Z	10.21	0.00 *	1.00	−12.74	−8.51
FCI DA-L	1.97	82.81	13.93	13.50	0.05	1.51	0.19	0.18	Z	10.21	0.00 *	1.00	−11.14	−8.64
FCI DA tot	1.91	59.96	13.48	10.42	0.03	0.86	0.18	0.11	Z	10.21	0.00 *	1.00	−12.17	−9.55
FCSI TA	−86.57	66.08	2.63	30.73	−47.26	82.69	−0.17	24.30	T	0.68	0.50	0.07	−12.06	6.47
FCSI MM	−46.28	63.44	5.59	25.66	−58.70	68.68	−0.76	24.42	Z	1.50	0.14	0.25	−13.55	4.35
FCSI SCM	−1048.60	11,407.25	240.19	1439.96	−77.67	44.46	3.82	21.27	Z	2.92	0.00 *	0.11	−60.62	−12.75
FCSI DA	−87.75	35.52	−1.00	21.27	−85.42	39.99	−1.29	24.43	T	0.08	0.94	0.01	−7.95	7.36
FCAI-R	−67.24	81.20	5.22	34.23	−81.87	71.72	−6.92	34.47	T	2.09	0.04 *	0.35	−25.61	−0.90
FCAI-L	−72.04	68.19	2.36	36.68	−68.18	63.36	−5.87	33.47	T	1.39	0.17	0.23	−21.03	3.60
FCAI tot	−63.98	73.90	4.57	31.66	−70.17	63.09	−7.08	30.90	T	2.22	0.03 *	0.22	−22.10	−1.19

Z—Mann–Whitney U test; T—Student’s *t*-test; ES—effect size; CI—confidence interval; Min.—minimum; Max.—maximum; SD—standard deviation; FCI—Functional Clenching Index; FCSI—Functional Clenching Symmetry Index; FCAI—Functional Clenching Activity Index; TA—temporalis muscle; MM—masseter muscle; SCM—sternocleidomastoid muscle; DA—digastric muscle; R—right side; L—left side; tot—both-sided; *—significant difference.

**Table 5:** Comparison of asymmetry and activity indices at rest and during maximal tooth clenching with and without stabilization splint.

Activity	Indices	Without Stabilization Splint				With Stabilization Splint				Test		*p*	ES	CI 95%	
		Min.	Max.	Mean	SD	Min.	Max.	Mean	SD						
Rest	ASI TA	−64.44	54.61	−1.86	25.92	−45.30	53.56	1.56	21.83	Z	−0.71	0.48	0.07	−5.31	11.16
	ASI MM	−60.83	35.71	−2.76	17.75	−69.71	60.03	3.04	24.02	T	−1.62	0.11	0.28	−1.26	12.86
	ASI SCM	−37.69	33.33	−5.48	13.82	−35.40	46.97	−2.55	17.04	T	−0.79	0.43	0.08	−2.26	8.11
	ASI DA	−18.22	28.70	1.13	9.66	−37.86	37.13	−0.89	13.99	T	0.75	0.45	0.07	−6.04	2.00
	ACI-R	−77.89	68.06	−5.14	31.65	−49.32	80.16	16.81	33.47	T	−3.99	0.00 *	0.67	11.07	32.85
	ACI-L	−76.70	53.92	−4.48	32.51	−54.25	80.43	15.48	33.49	T	−3.58	0.00 *	0.60	8.93	30.99
	ACI tot	−71.79	58.97	−5.82	29.78	−38.96	78.43	16.86	29.80	T	−3.95	0.00 *	0.39	12.73	32.64
Clenching	ASI TA	−74.21	69.31	0.50	18.49	−29.00	81.06	1.47	16.11	Z	−0.10	0.92	0.01	−4.52	5.15
	ASI MM	−32.62	45.74	3.15	16.78	−25.95	32.04	2.12	14.45	T	0.39	0.70	0.07	−6.26	4.20
	ASI SCM	−47.22	47.95	2.28	18.35	−81.14	51.49	1.44	21.23	Z	0.26	0.80	0.91	−6.41	5.54
	ASI DA	−88.11	35.09	0.07	21.73	−33.75	36.90	−0.01	19.41	Z	0.31	0.76	1.00	−7.87	5.85
	ACI-R	−47.73	79.27	−0.11	24.53	−84.60	55.88	11.79	24.14	T	−2.89	0.00 *	0.49	3.77	20.04
	ACI-L	−68.62	77.06	−2.64	24.34	−33.77	79.76	10.97	19.59	T	−3.65	0.00 *	0.62	6.23	21.00
	ACI tot	−44.63	51.76	−1.45	20.87	−72.57	67.71	11.27	20.63	T	−3.75	0.00 *	0.37	5.78	19.65

Z—Mann–Whitney U test; T—Student’s *t*-test; ES—effect size; CI—confidence interval; Min.—minimum; Max.—maximum; SD—standard deviation; ASI—Asymmetry Index; ACI—Activity Index; TA—temporalis muscle; MM— masseter muscle; SCM—sternocleidomastoid muscle; DA—digastric muscle; R—right side; L—left side; tot—both-sided; *—significant difference.

## Data Availability

The data presented in this study are available upon request from the corresponding author.
